# 3,4-Dihydroxy­benzaldehyde thio­semi­carbazone

**DOI:** 10.1107/S160053680801386X

**Published:** 2008-05-14

**Authors:** Kong Wai Tan, Yang Farina, Chew Hee Ng, Mohd Jamil Maah, Seik Weng Ng

**Affiliations:** a Department of Chemistry, University of Malaya, 50603 Kuala Lumpur, Malaysia; b School of Chemical Science and Food Technology, Universiti Kebangsaan Malaysia, 43600 Bangi, Malaysia; c Faculty of Engineering and Science, Universiti Tunku Abdul Rahman, 53300 Kuala Lumpur, Malaysia

## Abstract

The asymmetric unit of the title compound, C_8_H_9_N_3_O_2_S, contains three independent mol­ecules which are stacked approximately over each other. In the crystal structure, centrosymmetric pairs of mol­ecules are formed through inter­molecular hydr­oxy–hydr­oxy O—H⋯O and hydr­oxy–sulfur O—H⋯S hydrogen bonds which are, in turn, linked into a two-dimensional network by N—H⋯O(hydr­oxy) hydrogen bonds.

## Related literature

For the structure of 3,4-dihydroxy­benzaldehyde 4-phenyl­thio­semicarbazone, see: Swesi *et al.* (2006 ▶). For some metal complexes of the ligand, see: Zhu *et al.* (1991 ▶, 1997 ▶).
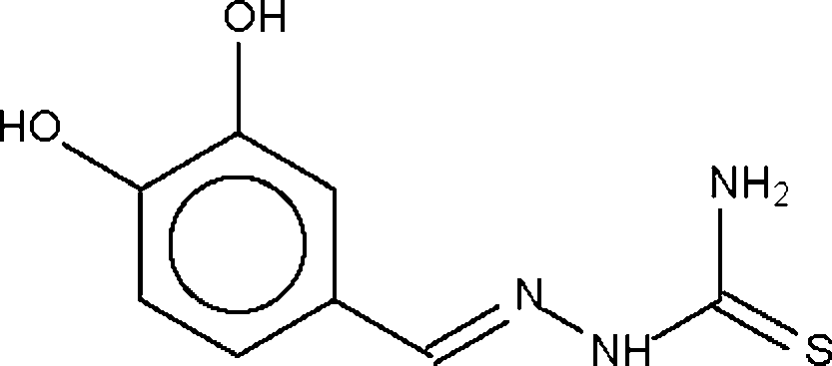


## Experimental

### 

#### Crystal data

C_8_H_9_N_3_O_2_S
*M* *_r_* = 211.24
Triclinic, 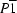

*a* = 10.657 (2) Å
*b* = 11.794 (2) Å
*c* = 12.356 (2) Å
α = 111.657 (2)°
β = 104.082 (2)°
γ = 90.929 (2)°
*V* = 1390.2 (4) Å^3^
*Z* = 6
Mo *K*α radiation
μ = 0.33 mm^−1^
*T* = 100 (2) K
0.20 × 0.18 × 0.04 mm

#### Data collection

Bruker SMART APEX diffractometer
Absorption correction: multi-scan (*SADABS*; Sheldrick, 1996 ▶) *T*_min_ = 0.938, *T*_max_ = 0.987
8792 measured reflections
6298 independent reflections
3727 reflections with *I* > 2σ(*I*)
*R*_int_ = 0.028

#### Refinement

*R*[*F* ^2^ > 2σ(*F* ^2^)] = 0.061
*wR*(*F* ^2^) = 0.189
*S* = 1.01
6298 reflections
397 parameters
6 restraints
H atoms treated by a mixture of independent and constrained refinement
Δρ_max_ = 0.51 e Å^−3^
Δρ_min_ = −0.40 e Å^−3^

### 

Data collection: *APEX2* (Bruker, 2007 ▶); cell refinement: *SAINT* (Bruker, 2007 ▶); data reduction: *SAINT*; program(s) used to solve structure: *SHELXS97* (Sheldrick, 2008 ▶); program(s) used to refine structure: *SHELXL97* (Sheldrick, 2008 ▶); molecular graphics: *X-SEED* (Barbour, 2001 ▶); software used to prepare material for publication: *publCIF* (Westrip, 2008 ▶).

## Supplementary Material

Crystal structure: contains datablocks I, global. DOI: 10.1107/S160053680801386X/lh2626sup1.cif

Structure factors: contains datablocks I. DOI: 10.1107/S160053680801386X/lh2626Isup2.hkl

Additional supplementary materials:  crystallographic information; 3D view; checkCIF report

## Figures and Tables

**Table 1:** Hydrogen-bond geometry (Å, °)

*D*—H⋯*A*	*D*—H	H⋯*A*	*D*⋯*A*	*D*—H⋯*A*
O1—H1o⋯O6^i^	0.84 (1)	2.07 (3)	2.784 (3)	143 (4)
O2—H2o⋯S1^ii^	0.84 (1)	2.47 (1)	3.300 (2)	171 (4)
N1—H1n2⋯O5^iii^	0.88	2.00	2.856 (4)	163
O3—H3o⋯O4^i^	0.84 (1)	2.11 (4)	2.732 (3)	130 (4)
O4—H4o⋯S2^ii^	0.84 (1)	2.38 (1)	3.219 (2)	174 (4)
N4—H4n2⋯O3^iii^	0.88	2.05	2.900 (4)	162
O5—H5o⋯O2^i^	0.84 (1)	2.16 (4)	2.742 (3)	127 (4)
O6—H6o⋯S3^ii^	0.84 (1)	2.40 (1)	3.244 (2)	177 (4)
N7—H7n2⋯O1^iii^	0.88	2.13	2.981 (4)	161

Symmetry codes: (i) 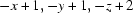; (ii) 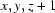; (iii) 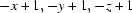.

**Fig. 1.**
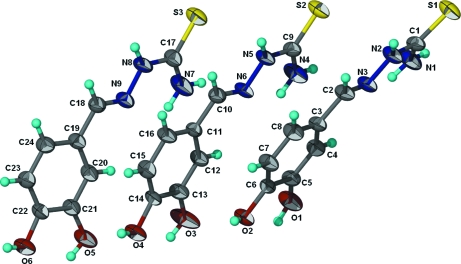
Thermal ellipsoid (Barbour, 2001) plot of the three independent molecules of C14H13N3O2S at the 70% probability level. Hydrogen atoms are drawn as spheres of arbitrary radii.

## supplementary crystallographic information

### Comment

A previous study of Schiff bases derived by condensing substituted benzaldehydes with 4-phenylthiosemicarbazides describes the 3,4-dihydroxybenzaldehyde derivative, which crystallizes as a hemihydrate. The compound features extensive hydrogen bonds (Swesi *et al.*, 2006). The condensation product of the reaction between thiosemicarbazide and 3,4-dihydroxybenzaldehyde has an amino –NH_2_ group in place of the phenyl group. In the crystal structure, a molecule is linked to an adjacent molecule by a hydrogen bond [*O*–H_3-hydroxy_···O_4-hydroxy_]; it is linked to another adjacent molecule by another hydrogen bond [*O*–H_4-hydroxy_···S]. The structure is consolidated into a two-dimensional network motif by a N_terminal_–*H*···O_4-hydroxy_ hydrogen bond. The asymmetric unit features three molecules that are approximately stacked over each other (Fig. 1).

### Experimental

Thiosemicarbazide (0.09 g, 1 mmol) and 2,4-dihydroxybenzaldehyde (0.14 g, 1 mmol) were heated in an ethanol/water mixture (20/5 ml) for 3 h. Slow evaporation of the solvent yielded yellow crystals.

### Refinement

Carbon-bound H-atoms were placed in calculated positions (C—H 0.95 Å) and were included in the refinement in the riding model approximation, with *U*(H) set to 1.2 *U*(C). The amino H-atoms were similarly treated (N–H 0.88 Å). The hydroxy H-atoms were located in a difference Fourier map, and were refined with a distance retraint of O–H 0.85±0.01 Å; their temperature factors were tied by a factor of 1.5.

### Crystal data

**Table:**

C_8_H_9_N_3_O_2_S	*Z* = 6
*M**_r_* = 211.24	*F*_000_ = 660
Triclinic, *P*1	*D*_x_ = 1.514 Mg m^−^^3^
Hall symbol: -P 1	Mo *K*α radiation λ = 0.71073 Å
*a* = 10.657 (2) Å	Cell parameters from 1478 reflections
*b* = 11.794 (2) Å	θ = 2.7–27.8º
*c* = 12.356 (2) Å	µ = 0.33 mm^−^^1^
α = 111.657 (2)º	*T* = 100 (2) K
β = 104.082 (2)º	Block, yellow
γ = 90.929 (2)º	0.20 × 0.18 × 0.04 mm
*V* = 1390.2 (4) Å^3^	

### Data collection

**Table:**

Bruker SMART APEX diffractometer	6298 independent reflections
Radiation source: fine-focus sealed tube	3727 reflections with *I* > 2σ(*I*)
Monochromator: graphite	*R*_int_ = 0.028
*T* = 100(2) K	θ_max_ = 27.5º
ω scans	θ_min_ = 1.8º
Absorption correction: multi-scan(SADABS; Sheldrick, 1996)	*h* = −13→13
*T*_min_ = 0.938, *T*_max_ = 0.987	*k* = −15→9
8792 measured reflections	*l* = −14→16

### Refinement

**Table:**

Refinement on *F*^2^	Secondary atom site location: difference Fourier map
Least-squares matrix: full	Hydrogen site location: inferred from neighbouring sites
*R*[*F*^2^ > 2σ(*F*^2^)] = 0.061	H atoms treated by a mixture of independent and constrained refinement
*wR*(*F*^2^) = 0.189	*w* = 1/[σ^2^(*F*_o_^2^) + (0.1004*P*)^2^] where *P* = (*F*_o_^2^ + 2*F*_c_^2^)/3
*S* = 1.01	(Δ/σ)_max_ = 0.001
6298 reflections	Δρ_max_ = 0.51 e Å^−^^3^
397 parameters	Δρ_min_ = −0.39 e Å^−^^3^
6 restraints	Extinction correction: none
Primary atom site location: structure-invariant direct methods	

### Fractional atomic coordinates and isotropic or equivalent isotropic displacement parameters (Å^2^)

**Table:**

	*x*	*y*	*z*	*U*_iso_*/*U*_eq_	
S1	0.84547 (10)	0.94314 (8)	0.21803 (8)	0.0401 (3)	
S2	0.45653 (10)	0.85179 (8)	0.31556 (7)	0.0359 (2)	
S3	0.10237 (10)	0.74686 (8)	0.41416 (8)	0.0390 (3)	
O1	0.7414 (3)	0.5896 (2)	0.7057 (2)	0.0440 (7)	
H1O	0.750 (4)	0.566 (4)	0.763 (3)	0.066*	
O2	0.7519 (2)	0.7405 (2)	0.9368 (2)	0.0333 (6)	
H2O	0.769 (4)	0.797 (3)	1.0057 (17)	0.050*	
O3	0.4715 (4)	0.5066 (2)	0.8312 (2)	0.0615 (9)	
H3O	0.476 (5)	0.496 (5)	0.895 (3)	0.092*	
O4	0.4659 (3)	0.6536 (2)	1.05646 (19)	0.0359 (6)	
H4O	0.464 (4)	0.710 (3)	1.122 (2)	0.054*	
O5	0.2270 (3)	0.4329 (2)	0.9629 (2)	0.0441 (7)	
H5O	0.232 (4)	0.426 (4)	1.029 (2)	0.066*	
O6	0.1658 (3)	0.5600 (2)	1.1704 (2)	0.0370 (6)	
H6O	0.152 (4)	0.609 (3)	1.235 (2)	0.056*	
N1	0.7685 (3)	0.7537 (3)	0.2628 (2)	0.0407 (8)	
H1N1	0.7486	0.7210	0.3109	0.049*	
H1N2	0.7622	0.7072	0.1868	0.049*	
N2	0.8145 (3)	0.9368 (2)	0.4209 (2)	0.0328 (7)	
H2N	0.8332	1.0172	0.4520	0.039*	
N3	0.7922 (3)	0.8755 (2)	0.4912 (2)	0.0316 (6)	
N4	0.4570 (3)	0.6598 (3)	0.3786 (3)	0.0438 (8)	
H4N1	0.4531	0.6259	0.4305	0.053*	
H4N2	0.4637	0.6141	0.3061	0.053*	
N5	0.4424 (3)	0.8421 (2)	0.5209 (2)	0.0294 (6)	
H5N	0.4379	0.9218	0.5460	0.035*	
N6	0.4390 (3)	0.7808 (2)	0.5960 (2)	0.0282 (6)	
N7	0.1429 (3)	0.5636 (3)	0.4892 (3)	0.0470 (9)	
H7N1	0.1457	0.5309	0.5431	0.056*	
H7N2	0.1607	0.5210	0.4205	0.056*	
N8	0.0866 (3)	0.7341 (3)	0.6182 (2)	0.0334 (7)	
H8N	0.0617	0.8082	0.6370	0.040*	
N9	0.1000 (3)	0.6760 (2)	0.6982 (2)	0.0299 (6)	
C1	0.8075 (3)	0.8720 (3)	0.3043 (3)	0.0300 (7)	
C2	0.7942 (3)	0.9415 (3)	0.5994 (3)	0.0306 (7)	
H2	0.8072	1.0284	0.6258	0.037*	
C3	0.7772 (3)	0.8876 (3)	0.6840 (3)	0.0279 (7)	
C4	0.7636 (3)	0.7614 (3)	0.6542 (3)	0.0296 (7)	
H4	0.7605	0.7070	0.5743	0.035*	
C5	0.7545 (3)	0.7147 (3)	0.7389 (3)	0.0293 (7)	
C6	0.7597 (3)	0.7934 (3)	0.8565 (3)	0.0257 (7)	
C7	0.7714 (3)	0.9180 (3)	0.8871 (3)	0.0319 (8)	
H7C	0.7737	0.9719	0.9670	0.038*	
C8	0.7799 (3)	0.9652 (3)	0.8013 (3)	0.0327 (8)	
H8	0.7875	1.0516	0.8228	0.039*	
C9	0.4526 (3)	0.7787 (3)	0.4095 (3)	0.0273 (7)	
C10	0.4204 (3)	0.8443 (3)	0.6983 (3)	0.0288 (7)	
H10	0.4054	0.9277	0.7164	0.035*	
C11	0.4218 (3)	0.7917 (3)	0.7876 (3)	0.0252 (7)	
C12	0.4409 (3)	0.6698 (3)	0.7657 (3)	0.0298 (7)	
H12	0.4459	0.6164	0.6879	0.036*	
C13	0.4526 (3)	0.6261 (3)	0.8557 (3)	0.0321 (8)	
C14	0.4480 (3)	0.7039 (3)	0.9711 (3)	0.0257 (7)	
C15	0.4243 (3)	0.8234 (3)	0.9921 (3)	0.0316 (8)	
H15	0.4172	0.8760	1.0694	0.038*	
C16	0.4107 (3)	0.8671 (3)	0.9006 (3)	0.0304 (7)	
H16	0.3936	0.9495	0.9153	0.036*	
C17	0.1122 (3)	0.6761 (3)	0.5111 (3)	0.0306 (7)	
C18	0.0760 (3)	0.7363 (3)	0.7984 (3)	0.0286 (7)	
H18	0.0465	0.8147	0.8120	0.034*	
C19	0.0928 (3)	0.6872 (3)	0.8926 (3)	0.0264 (7)	
C20	0.1458 (3)	0.5772 (3)	0.8812 (3)	0.0288 (7)	
H20	0.1662	0.5295	0.8081	0.035*	
C21	0.1686 (3)	0.5376 (3)	0.9745 (3)	0.0291 (7)	
C22	0.1375 (3)	0.6051 (3)	1.0814 (3)	0.0270 (7)	
C23	0.0804 (3)	0.7116 (3)	1.0922 (3)	0.0295 (7)	
H23	0.0556	0.7566	1.1637	0.035*	
C24	0.0594 (3)	0.7524 (3)	0.9982 (3)	0.0300 (7)	
H24	0.0214	0.8264	1.0064	0.036*	

### Atomic displacement parameters (Å^2^)

**Table:**

	*U*^11^	*U*^22^	*U*^33^	*U*^12^	*U*^13^	*U*^23^
S1	0.0718 (7)	0.0287 (5)	0.0288 (5)	0.0104 (4)	0.0234 (4)	0.0146 (4)
S2	0.0636 (6)	0.0242 (4)	0.0253 (4)	0.0079 (4)	0.0172 (4)	0.0123 (4)
S3	0.0631 (6)	0.0330 (5)	0.0286 (5)	0.0124 (4)	0.0187 (4)	0.0164 (4)
O1	0.083 (2)	0.0205 (12)	0.0354 (14)	0.0112 (13)	0.0242 (14)	0.0123 (11)
O2	0.0527 (15)	0.0269 (13)	0.0263 (12)	0.0067 (11)	0.0144 (11)	0.0144 (10)
O3	0.137 (3)	0.0278 (15)	0.0344 (15)	0.0361 (17)	0.0385 (18)	0.0173 (13)
O4	0.0637 (17)	0.0257 (13)	0.0238 (12)	0.0131 (12)	0.0166 (12)	0.0123 (10)
O5	0.083 (2)	0.0266 (13)	0.0343 (14)	0.0200 (13)	0.0278 (14)	0.0167 (12)
O6	0.0633 (17)	0.0276 (13)	0.0252 (12)	0.0146 (12)	0.0162 (12)	0.0127 (11)
N1	0.075 (2)	0.0255 (16)	0.0245 (15)	0.0053 (15)	0.0198 (15)	0.0091 (13)
N2	0.0565 (19)	0.0214 (14)	0.0249 (14)	0.0070 (13)	0.0161 (13)	0.0105 (12)
N3	0.0492 (18)	0.0255 (15)	0.0270 (14)	0.0069 (13)	0.0133 (13)	0.0156 (12)
N4	0.085 (3)	0.0232 (16)	0.0285 (16)	0.0122 (16)	0.0230 (16)	0.0106 (13)
N5	0.0500 (18)	0.0194 (13)	0.0235 (14)	0.0081 (12)	0.0140 (12)	0.0107 (11)
N6	0.0444 (17)	0.0221 (14)	0.0230 (13)	0.0032 (12)	0.0105 (12)	0.0131 (11)
N7	0.088 (3)	0.0285 (17)	0.0371 (17)	0.0156 (17)	0.0319 (17)	0.0165 (14)
N8	0.0529 (19)	0.0271 (15)	0.0302 (15)	0.0141 (13)	0.0172 (13)	0.0179 (13)
N9	0.0426 (17)	0.0239 (14)	0.0293 (14)	0.0056 (12)	0.0133 (12)	0.0147 (12)
C1	0.043 (2)	0.0262 (17)	0.0261 (17)	0.0123 (15)	0.0121 (15)	0.0143 (15)
C2	0.044 (2)	0.0221 (16)	0.0274 (17)	0.0042 (15)	0.0093 (15)	0.0119 (14)
C3	0.0387 (19)	0.0247 (17)	0.0249 (16)	0.0075 (14)	0.0106 (14)	0.0134 (14)
C4	0.042 (2)	0.0221 (16)	0.0261 (17)	0.0081 (14)	0.0122 (14)	0.0087 (14)
C5	0.0412 (19)	0.0190 (16)	0.0316 (17)	0.0082 (14)	0.0128 (15)	0.0119 (14)
C6	0.0326 (18)	0.0267 (17)	0.0235 (16)	0.0092 (14)	0.0116 (13)	0.0132 (14)
C7	0.050 (2)	0.0246 (17)	0.0223 (16)	0.0063 (15)	0.0118 (15)	0.0086 (14)
C8	0.049 (2)	0.0179 (16)	0.0316 (18)	0.0037 (15)	0.0121 (16)	0.0094 (14)
C9	0.0363 (18)	0.0234 (17)	0.0245 (16)	0.0080 (14)	0.0089 (14)	0.0111 (14)
C10	0.0357 (19)	0.0256 (17)	0.0293 (17)	0.0079 (14)	0.0114 (14)	0.0137 (14)
C11	0.0315 (17)	0.0240 (16)	0.0250 (16)	0.0059 (13)	0.0099 (13)	0.0133 (14)
C12	0.046 (2)	0.0250 (17)	0.0211 (15)	0.0092 (15)	0.0134 (14)	0.0092 (14)
C13	0.050 (2)	0.0209 (16)	0.0306 (17)	0.0120 (15)	0.0156 (15)	0.0126 (14)
C14	0.0371 (18)	0.0245 (16)	0.0211 (15)	0.0062 (14)	0.0111 (13)	0.0128 (13)
C15	0.047 (2)	0.0236 (17)	0.0259 (17)	0.0085 (15)	0.0163 (15)	0.0074 (14)
C16	0.048 (2)	0.0191 (16)	0.0296 (17)	0.0078 (14)	0.0133 (15)	0.0132 (14)
C17	0.0397 (19)	0.0259 (17)	0.0288 (17)	0.0040 (15)	0.0122 (15)	0.0116 (15)
C18	0.0333 (18)	0.0267 (17)	0.0289 (17)	0.0061 (14)	0.0096 (14)	0.0131 (14)
C19	0.0337 (18)	0.0221 (16)	0.0242 (16)	0.0019 (13)	0.0079 (13)	0.0098 (13)
C20	0.042 (2)	0.0212 (16)	0.0262 (16)	0.0048 (14)	0.0140 (14)	0.0094 (14)
C21	0.0408 (19)	0.0205 (16)	0.0300 (17)	0.0070 (14)	0.0141 (14)	0.0111 (14)
C22	0.0361 (18)	0.0240 (16)	0.0242 (16)	0.0036 (14)	0.0103 (14)	0.0115 (14)
C23	0.0405 (19)	0.0252 (17)	0.0245 (16)	0.0092 (15)	0.0131 (14)	0.0084 (14)
C24	0.0375 (19)	0.0248 (17)	0.0281 (17)	0.0067 (14)	0.0089 (14)	0.0103 (14)

### Geometric parameters (Å, °)

**Table:**

S1—C1	1.693 (3)	N9—C18	1.270 (4)
S2—C9	1.689 (3)	C2—C3	1.453 (4)
S3—C17	1.680 (3)	C2—H2	0.9500
O1—C5	1.372 (4)	C3—C4	1.391 (4)
O1—H1O	0.837 (10)	C3—C8	1.393 (4)
O2—C6	1.369 (4)	C4—C5	1.372 (4)
O2—H2O	0.840 (10)	C4—H4	0.9500
O3—C13	1.358 (4)	C5—C6	1.392 (4)
O3—H3O	0.836 (10)	C6—C7	1.371 (4)
O4—C14	1.367 (4)	C7—C8	1.387 (4)
O4—H4O	0.841 (10)	C7—H7C	0.9500
O5—C21	1.368 (4)	C8—H8	0.9500
O5—H5O	0.838 (10)	C10—C11	1.450 (4)
O6—C22	1.364 (4)	C10—H10	0.9500
O6—H6O	0.844 (10)	C11—C16	1.385 (4)
N1—C1	1.316 (4)	C11—C12	1.390 (4)
N1—H1N1	0.8800	C12—C13	1.369 (4)
N1—H1N2	0.8800	C12—H12	0.9500
N2—C1	1.341 (4)	C13—C14	1.396 (4)
N2—N3	1.375 (3)	C14—C15	1.375 (4)
N2—H2N	0.8800	C15—C16	1.385 (4)
N3—C2	1.269 (4)	C15—H15	0.9500
N4—C9	1.316 (4)	C16—H16	0.9500
N4—H4N1	0.8800	C18—C19	1.457 (4)
N4—H4N2	0.8800	C18—H18	0.9500
N5—C9	1.335 (4)	C19—C24	1.384 (4)
N5—N6	1.377 (3)	C19—C20	1.397 (4)
N5—H5N	0.8800	C20—C21	1.368 (4)
N6—C10	1.276 (4)	C20—H20	0.9500
N7—C17	1.316 (4)	C21—C22	1.390 (4)
N7—H7N1	0.8800	C22—C23	1.380 (4)
N7—H7N2	0.8800	C23—C24	1.386 (4)
N8—C17	1.344 (4)	C23—H23	0.9500
N8—N9	1.380 (3)	C24—H24	0.9500
N8—H8N	0.8800		
			
C5—O1—H1O	115 (3)	C3—C8—H8	119.6
C6—O2—H2O	107 (3)	N4—C9—N5	116.7 (3)
C13—O3—H3O	106 (4)	N4—C9—S2	123.5 (2)
C14—O4—H4O	107 (3)	N5—C9—S2	119.8 (2)
C21—O5—H5O	103 (3)	N6—C10—C11	121.2 (3)
C22—O6—H6O	112 (3)	N6—C10—H10	119.4
C1—N1—H1N1	120.0	C11—C10—H10	119.4
C1—N1—H1N2	120.0	C16—C11—C12	119.0 (3)
H1N1—N1—H1N2	120.0	C16—C11—C10	118.9 (3)
C1—N2—N3	118.9 (3)	C12—C11—C10	122.0 (3)
C1—N2—H2N	120.5	C13—C12—C11	120.3 (3)
N3—N2—H2N	120.5	C13—C12—H12	119.8
C2—N3—N2	116.4 (3)	C11—C12—H12	119.8
C9—N4—H4N1	120.0	O3—C13—C12	118.8 (3)
C9—N4—H4N2	120.0	O3—C13—C14	120.7 (3)
H4N1—N4—H4N2	120.0	C12—C13—C14	120.5 (3)
C9—N5—N6	118.9 (3)	O4—C14—C15	124.2 (3)
C9—N5—H5N	120.6	O4—C14—C13	116.4 (3)
N6—N5—H5N	120.6	C15—C14—C13	119.4 (3)
C10—N6—N5	116.1 (3)	C14—C15—C16	120.0 (3)
C17—N7—H7N1	120.0	C14—C15—H15	120.0
C17—N7—H7N2	120.0	C16—C15—H15	120.0
H7N1—N7—H7N2	120.0	C15—C16—C11	120.6 (3)
C17—N8—N9	119.0 (3)	C15—C16—H16	119.7
C17—N8—H8N	120.5	C11—C16—H16	119.7
N9—N8—H8N	120.5	N7—C17—N8	115.9 (3)
C18—N9—N8	115.9 (3)	N7—C17—S3	124.0 (3)
N1—C1—N2	116.6 (3)	N8—C17—S3	120.0 (3)
N1—C1—S1	123.3 (2)	N9—C18—C19	121.1 (3)
N2—C1—S1	120.1 (2)	N9—C18—H18	119.5
N3—C2—C3	121.7 (3)	C19—C18—H18	119.5
N3—C2—H2	119.2	C24—C19—C20	118.6 (3)
C3—C2—H2	119.2	C24—C19—C18	119.6 (3)
C4—C3—C8	118.5 (3)	C20—C19—C18	121.8 (3)
C4—C3—C2	122.7 (3)	C21—C20—C19	120.4 (3)
C8—C3—C2	118.8 (3)	C21—C20—H20	119.8
C5—C4—C3	120.7 (3)	C19—C20—H20	119.8
C5—C4—H4	119.7	O5—C21—C20	119.0 (3)
C3—C4—H4	119.7	O5—C21—C22	120.3 (3)
O1—C5—C4	118.7 (3)	C20—C21—C22	120.6 (3)
O1—C5—C6	121.0 (3)	O6—C22—C23	123.8 (3)
C4—C5—C6	120.3 (3)	O6—C22—C21	116.6 (3)
O2—C6—C7	123.0 (3)	C23—C22—C21	119.6 (3)
O2—C6—C5	117.1 (3)	C22—C23—C24	119.5 (3)
C7—C6—C5	119.9 (3)	C22—C23—H23	120.2
C6—C7—C8	119.8 (3)	C24—C23—H23	120.2
C6—C7—H7C	120.1	C19—C24—C23	121.2 (3)
C8—C7—H7C	120.1	C19—C24—H24	119.4
C7—C8—C3	120.8 (3)	C23—C24—H24	119.4
C7—C8—H8	119.6		
			
C1—N2—N3—C2	−176.9 (3)	C11—C12—C13—C14	−1.3 (5)
C9—N5—N6—C10	−175.5 (3)	O3—C13—C14—O4	1.2 (5)
C17—N8—N9—C18	179.2 (3)	C12—C13—C14—O4	−177.4 (3)
N3—N2—C1—N1	5.5 (5)	O3—C13—C14—C15	−177.8 (3)
N3—N2—C1—S1	−175.4 (2)	C12—C13—C14—C15	3.7 (5)
N2—N3—C2—C3	−177.5 (3)	O4—C14—C15—C16	178.4 (3)
N3—C2—C3—C4	2.9 (5)	C13—C14—C15—C16	−2.7 (5)
N3—C2—C3—C8	−179.7 (3)	C14—C15—C16—C11	−0.5 (5)
C8—C3—C4—C5	−0.8 (5)	C12—C11—C16—C15	2.9 (5)
C2—C3—C4—C5	176.6 (3)	C10—C11—C16—C15	−173.6 (3)
C3—C4—C5—O1	−179.9 (3)	N9—N8—C17—N7	3.0 (5)
C3—C4—C5—C6	−0.4 (5)	N9—N8—C17—S3	−177.6 (2)
O1—C5—C6—O2	0.4 (5)	N8—N9—C18—C19	−177.0 (3)
C4—C5—C6—O2	−179.1 (3)	N9—C18—C19—C24	−176.7 (3)
O1—C5—C6—C7	−179.3 (3)	N9—C18—C19—C20	5.3 (5)
C4—C5—C6—C7	1.2 (5)	C24—C19—C20—C21	−2.5 (5)
O2—C6—C7—C8	179.5 (3)	C18—C19—C20—C21	175.5 (3)
C5—C6—C7—C8	−0.9 (5)	C19—C20—C21—O5	−176.6 (3)
C6—C7—C8—C3	−0.3 (5)	C19—C20—C21—C22	1.1 (5)
C4—C3—C8—C7	1.1 (5)	O5—C21—C22—O6	−1.2 (5)
C2—C3—C8—C7	−176.4 (3)	C20—C21—C22—O6	−178.8 (3)
N6—N5—C9—N4	0.9 (5)	O5—C21—C22—C23	179.0 (3)
N6—N5—C9—S2	179.8 (2)	C20—C21—C22—C23	1.4 (5)
N5—N6—C10—C11	−176.7 (3)	O6—C22—C23—C24	177.8 (3)
N6—C10—C11—C16	175.3 (3)	C21—C22—C23—C24	−2.5 (5)
N6—C10—C11—C12	−1.1 (5)	C20—C19—C24—C23	1.4 (5)
C16—C11—C12—C13	−2.0 (5)	C18—C19—C24—C23	−176.6 (3)
C10—C11—C12—C13	174.4 (3)	C22—C23—C24—C19	1.0 (5)
C11—C12—C13—O3	−179.9 (3)		

### Hydrogen-bond geometry (Å, °)

**Table:**

*D*—H···*A*	*D*—H	H···*A*	*D*···*A*	*D*—H···*A*
O1—H1o···O6^i^	0.84 (1)	2.07 (3)	2.784 (3)	143 (4)
O2—H2o···S1^ii^	0.84 (1)	2.47 (1)	3.300 (2)	171 (4)
N1—H1n2···O5^iii^	0.88	2.00	2.856 (4)	163
O3—H3o···O4^i^	0.84 (1)	2.11 (4)	2.732 (3)	130 (4)
O4—H4o···S2^ii^	0.84 (1)	2.38 (1)	3.219 (2)	174 (4)
N4—H4n2···O3^iii^	0.88	2.05	2.900 (4)	162
O5—H5o···O2^i^	0.84 (1)	2.16 (4)	2.742 (3)	127 (4)
O6—H6o···S3^ii^	0.84 (1)	2.40 (1)	3.244 (2)	177 (4)
N7—H7n2···O1^iii^	0.88	2.13	2.981 (4)	161

Symmetry codes: (i) −*x*+1, −*y*+1, −*z*+2; (ii) *x*, *y*, *z*+1; (iii) −*x*+1, −*y*+1, −*z*+1.
